# The Role of T Lymphocytes in the Pathogenesis of Paroxysmal Nocturnal Hemoglobinuria

**DOI:** 10.3389/fimmu.2021.777649

**Published:** 2021-12-24

**Authors:** Chenyuan Li, Xifeng Dong, Huaquan Wang, Zonghong Shao

**Affiliations:** Department of Hematology and Oncology, Tianjin Medical University General Hospital, Tianjin, China

**Keywords:** paroxysmal nocturnal hemoglobinuria, aplastic anemia, T lymphocytes, pathogenesis, immune escape

## Abstract

Paroxysmal nocturnal hemoglobinuria (PNH) is an acquired hematopoietic stem cell genetic mutation disease that causes defective erythrocyte membrane hemolysis. Its pathologic basis is the mutation of the *PIG-A* gene, whose product is necessary for the synthesis of glycosylphosphatidylinositol (GPI) anchors; the mutation of *PIG-A* gene results in the reduction or deletion of the GPI anchor, which leads to the deficiency of GPI-anchored proteins (GPI-APs), such as CD55 and CD59, which are complement inhibitors. The deficiency of complement inhibitors causes chronic complement-mediated intravascular hemolysis of GPI-anchor-deficient erythrocyte. *PIG-A* gene mutation could also be found in bone marrow hematopoietic stem cells (HSCs) of healthy people, but they have no growth advantage; only the HSCs with *PIG-A* gene mutation in PNH patients have this advantage and expand. Besides, HSCs from *PIG-A*-knockout mice do not show clonal expansion in bone marrow, so *PIG-A* mutation cannot explain the clonal advantage of the PNH clone and some additional factors are needed; thus, in recent years, many scholars have put forward the theories of the second hit, and immune escape theory is one of them. In this paper, we focus on how T lymphocytes are involved in immune escape hypothesis in the pathogenesis of PNH.

## Introduction

Paroxysmal nocturnal hemoglobinuria (PNH) is an acquired hematopoietic stem cell (HSC) genetic mutation disease, causing defective erythrocyte membrane hemolysis. It is a benign clonal disease, characterized by intravascular hemolysis, hemoglobinuria, venous thrombosis, and bone marrow failure (1). In 2007, the incidence of PNH was reported to be 1.59 per 100,000 people in the United Kingdom (2). According to a recent study, the incidence rate of PNH and aplastic anemia (AA)-PNH syndrome was about 0.35 cases per 100,000 people per year, and the overall prevalence rate was 3.81 per 100,000 (3). If not diagnosed or treated well, it can cause a 35% death rate within 5 years (4). Due to the low incidence rate of PNH, the disease may be ignored easily, which often leads to misdiagnoses and missed diagnoses.

**Figure 1:**
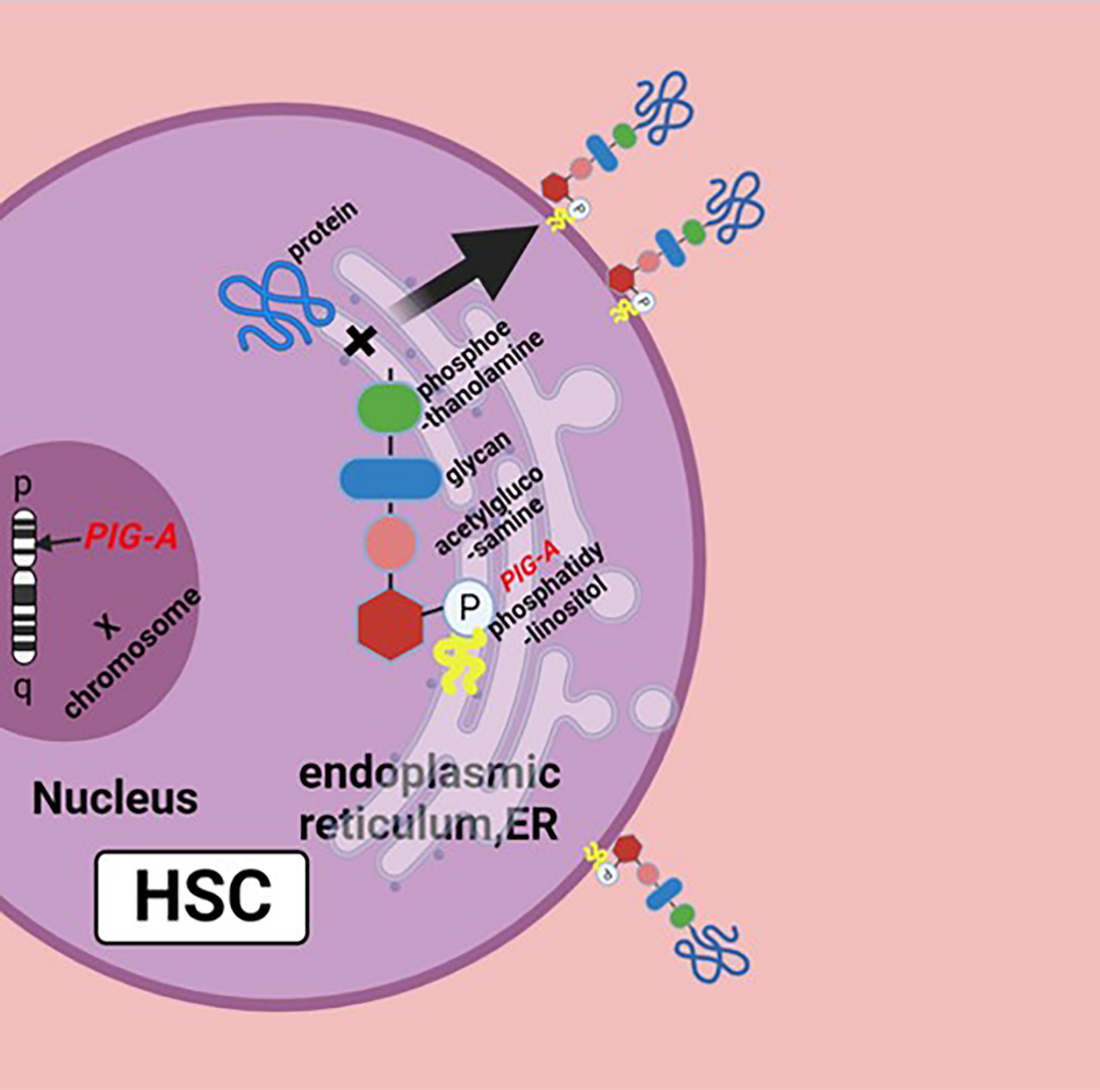
Biosynthesis of GPI molecules: *PIG-A* gene is located on chromosome Xp22.1, whose product is necessary for the synthesis of GPI. In endoplasmic reticulum (ER), the assembly of phosphatidylinositol, acetylglucosamine, and glycan consisting of three mannose molecules and a phosphoethanolamine is the first step of GPI synthesis. GPI and protein form a complex, then the GPI–protein complex transfers to the cell surface, and the GPI molecule becomes the anchor of the protein. The mutation of *PIG-A* gene causes the deficiency of GPIs and GPI-APs, or none at all. Figure was created in BioRender.com.

The pathophysiology of PNH is the genetic mutation of *Phosphatidylinositol glycan anchor biosynthesis, class A(PIG-A)* gene on chromosome Xp22.1, whose gene product is necessary for the synthesis of glycosylphosphatidylinositol (GPI) anchors; the reduction or deletion of the GPI anchor is the result of mutation of *PIG-A* gene, which leads to the deficiency of GPI-anchored proteins (GPI-APs) (**Figure 1**) (5). There are a large number of GPI-APs on the cell surface, including complement regulatory proteins like complement decay-accelerating factor (DAF, CD55) and membrane inhibitor of reactive lysis (MIRL, CD59), both of which are complement inhibitors; the primary function of CD55 is to dissociate and inactivate the C3 convertases, and CD59 prevents the formation of membrane attack complex (MAC or C5b–9 complex). The deficiency of these complement inhibitors causes chronic complement-mediated intravascular hemolysis of GPI-anchor-deficient erythrocyte (2, 6, 7). Early this century, the use of the anti-C5 antibody eculizumab has changed the management of PNH patients and may further improve their life (8). Noval complement inhibitors, like ravulizumab, a long-term C5 inhibitor, and pegcetacoplan, a C3 inhibitor, have also been now approved with PNH. New drugs like factor D and factor B inhibitors are in development (9, 10). Allogeneic hematopoietic stem cell transplantation (Allo-HSCT) is an option for PNH patients, which can effectively and safely eliminate the PNH clone with satisfactory overall survival (11).

*PIG-A* gene mutation could also be found in bone marrow hematopoietic stem cells (HSCs) of normal people, but they have no growth advantage under normal conditions; only the *PIG-A* gene mutation-HSCs in patients with PNH have this advantage, as well as clonal expansion (12), and HSCs from *PIG-A*-knockout mice do not show clonal expansion in the bone marrow (13), so *PIG-A* gene mutation alone cannot explain how the PNH clone can expand. Thus, in recent years, many scholars have put forward the theories of the second hit (2), and immune escape theory is one of them, which considers that HSCs expressing GPI-APs are killed by autoimmune cytotoxic lymphocytes (CTLs); however, GPI-APs-deficient HSCs can escape (14). This idea was first proposed 2 decades ago and is supported by several studies. Therefore, this paper focuses on how T lymphocytes are involved in immune escape hypothesis in the pathogenesis of PNH.

## T Lymphocytes’ Immunity Is Abnormal in PNH Patients

### Abnormal T lymphocytes’ Clones Were Found in Patients With PNH

Luzzatto L summarized their findings and then found that T-lymphocyte populations clonally expanded in many PNH patients (15). Fragments of peptides on major histocompatibility complex (MHC) molecules can activate T cells of the same MHC alleles. The subsequent cloning of the two chains of T-cell receptor (TCR), including α chain and β chain, which can specifically recognize the peptide/MHC, was found in the human body, supporting a model in the process of T-cell activation, in which proteins are digested and become short peptides, which combine with the MHC molecule, and then T cells of the same MHC alleles recognize these peptide/MHCs *via* the mutual effect between TCR and peptide/MHC. TCRs have three variable regions. Complementary determining region-3 (CDR-3) of the beta variable (Vβ) chain is one of them; as the main driver of recognition between TCR and peptide/MHC, it can regulate the specificity of the TCR/MHC interaction (16). T-cell activation requires two signals: TCR’s recognition of the MHC/peptide and the co-signal delivered by the interaction between the co-signaling molecule and its receptor. Risitano et al. found an overexpression of Vβ families in PNH and AA patients compared to normal control by Vβ cytometry (17). They used TCR flow cytometry and CDR-3 analysis to assess clonality of T lymphocytes and found that TCR-Vβ-specific expansions were detected in 10 PNH patients and 14 AA/PNH patients, and among four of them, extreme expansions of one Vβ-subset of CD8^+^/CD28^-^/CD56^+^(effector) phenotype were found, which is very similar to large granulocytic lymphocytic (LGL) leukemia (18); these lines of evidence support the idea that T lymphocyte is involved in the pathogenesis of PNH.

### PNH Is Closely Related to AA, Which Is an Autoimmune and Bone Marrow Destructive Disease

Clinical features of PNH include severe hemolysis, thrombosis, and cytopenia; bone marrow failure is regarded as its clinical manifestation, which is very similar to AA (19), and analysis of the databases found that about half of the PNH patients had a previous history of AA (20). Frickhofen et al. found in several clinical studies that Ham test results of AA patients turned positive after several months of immunosuppressive therapy (IST) with anti-thymocyte globin (ATG) and cyclosporine-A (CsA); it turns out that patients with AA may evolve into PNH (21). Colony analysis results of PNH patients were similar to those of AA patients (22). Some experts think that PNH is a unique subset of the AA. Increasing lines of evidence show that T lymphocytes cause HSC damage in AA patients and that the IST can overcome AA; therefore, autoimmune-mediated HSC destruction plays an important role in the pathogenesis of AA (23). Autoimmune response requires the participation of CD4^+,^CD8^+^ T cells, natural killer (NK) cells, and so on (24), and it is widely recognized that dysregulated CD4^+^ T cells, CD8^+^ T cells, and NK cells, and the production of various cytokines, such as interferon-gamma (IFN-γ), tumor necrosis factor-alpha (TNF-α), and transforming growth factor-beta (TGF-β), induce autoimmune-mediated apoptosis of HSCs in AA patients (25). All of these support the immune escape model of PNH, indicating that the autoimmunity in which T lymphocytes are involved in may play an important role in the pathogenesis of the disease.

### Immunosuppressive Therapy Is Effective in Some Patients With PNH

Kulagin et al. evaluated the influence that PNH clones may exert on 125 AA patients treated with IST; they were divided into two groups: the patients with the PNH clone group (PNH+ group) and the patients with no PNH clone group (PNH- group). After 6 months of IST, the response rate was higher in the PNH+ group than that in the PNH- group (26). Ren et al. found that the PNH clone predicts a faster response to IST in severe AA patients (27). ATG is an immunosuppressant that mainly acts on activated T lymphocytes, which can reduce the number of T lymphocytes. Seven PNH patients were treated with ATG in Paquette’s research, and three of them experienced improvement (28). Nakasone et al. applied ATG in the treatment of 4 patients with PNH; ATG was administered at a dose of 15 mg/kg and continued for 5 days. During the treatment, the patients showed aggravation of hemolysis and thrombocytopenia, and they all received blood transfusion of components such as RBCs and platelets, but no renal failure or thrombosis occurred during the therapy, and the anemia symptoms of the patients were all improved within 1 year (29). IST is effective in some PNH patients, further suggesting that the autoimmunity that T lymphocytes are involved in may play a role in the pathogenesis of the disease. However, Schubert et al. found that PNH clones appeared in some AA patients after IST (30); does that mean IST can drive PNH? Li et al. found in their study of 678 AA patients that, after IST, only 43 cases’ PNH clone switched from negative to positive and the PNH clone disappeared in 47 cases (31). Zhang et al. found that after IST, the PNH clone switched from negative to positive in 24 AA patients, remained positive PNH in 22 AA patients, and disappeared in 10 AA patients; these changes had no significant influence on overall responsive rates and survival rates (32). So far, IST is indeed effective for some PNH patients, but whether IST can drive PNH is still controversial.

## Differences Between GPI^-^ and GPI^+^ Cells in Patients With PNH

### The Proliferation Ability of GPI^-^ Cells and GPI^+^ Cells in Patients With PNH Is Different

Han Bing et al. found that, compared to PNH CD34^+^CD59^+^ cells, PNH CD34^+^CD59^-^ cells had a higher ability of plating efficiency, colony formation, and cell expansion (33). CD160 is a kind of GPI-AP, which is mainly expressed on the surface of some cells with cytotoxic activity, such as CD8^+^T, natural killer T (NKT) cells, and NK cells (34). As for the function of the CD160 molecule, it is recently believed that CD160 plays a co-inhibitory role by strongly binding to herpes virus entry mediator (HVEM) (35). CD160 inhibits proliferation of human T cells upon ligation to HVEM (36). Liu et al. separated CD160 ^+^(GPI^+^)CD8^+^ T cells and CD160^-^(GPI^-^) CD8^+^ T cells into two different groups, stimulated the cells in the two groups by IL-2, respectively, and observed the proliferation of cells in the two groups after 12, 24, 48, and 96 h; the results showed that the proliferation of cells in the two groups is approximately the same after 12 h and 24 h, but after 48 h and 96 h, the proliferation capacity of CD160^-^(GPI^-^) T cells is much higher than that of CD160^+^(GPI^+^) T cells, and the cytotoxicity mediated by CD160^-^(GPI^-^)CD8^+^ T cells was significantly higher than that mediated by CD160^+^(GPI^+^)CD8^+^ T cells (37). Katagiri et al. found that HVEM significantly inhibited the proliferation of GPI^+^ memory T cells, but it did not affect the proliferation of GPI^-^ memory T cells; in their point of view, memory T cells act like HSCs in some ways (38). The most possible explanation is that HSCs expressing some GPI-APs are very similar to memory T cells in Katagiri’s experiment, which express CD160; both of them may become invulnerable to some inhibitory proteins (e.g., HVEM) if they lack GPI-APs.

### The Apoptosis of GPI- Cells Differs From the Apoptosis of GPI+ Cells in Patients With PNH

Kunyaboon et al. found that CD59^+^ granulocytes showed more apoptosis than CD59^-^ granulocytes in PNH patients after being in the liquid growth culture system for 0 h and 4 h (39). FasL binds to Fas, which results in cell apoptosis (40). Ismall et al. found that the apoptosis rate of CD34^+^CD59^-^ cells was significantly lower than that of CD34^+^ CD59^+^ cells from the same PNH patient, and Fas expression was lower in CD59^-^ cells than that in CD59^+^ cells in 3 PNH patients (41). The CD34^+^/CD59^+^ cells in PNH patients seem to act similarly to the CD34^+^ cells in AA in terms of higher apoptosis with higher expression of Fas (42); however, the CD34^+^/CD59^−^ cells did not. Liu et al. separated CD160^+^(GPI^+^) CD8^+^ T cells and CD160^-^(GPI^-^) CD8^+^ T cells into two different groups and then stimulated cells in the two groups by IL-2; they found that after 48 h and 96 h, the apoptosis rate of CD160^+^(GPI^+^) T cells is much higher than that of CD160^-^(GPI^-^) T cells (37). These suggest a further hypothesis that GPI^-^ HSCs are resistant to apoptosis caused by cytokines, which may be related to the Fas/FasL pathway.

### The Relationship Between the Differences Mentioned Above and T Lymphocytes

Kunyaboon et al. found that CD8^+^ T lymphocytes inhibited CFU-GM and BFU-E colony formation of PNH patients (39). This suggests that T lymphocytes may play a role in the pathogenesis of PNH. Based on the immune escape hypothesis, Murakami et al. found that compared to mice without CD4^+^ T cells, ratios of fetal liver-derived cells in polymorphonuclear cells (PMNCs) and monocytes were smaller in CD4^+^ T cells co-transplanted mice; however, ratios of GPI^−^ cells in PMNCs and monocytes in CD4^+^ T cells co-transplanted mice were significantly increased, suggesting that GPI^+^ cells were selectively killed and GPI^-^ cells survived (14). Ikeda et al. found that the number of Wilms’ tumor gene (WT1) peptide-specific cytotoxic CD8^+^ T lymphocytes and the number of interferon (IFN)-γ-producing mononuclear cells (MNC) stimulated by WT1 in peripheral blood of 5 patients with PNH were significantly increased compared with 8 normal controls. WT1 peptide-specific and human leukocyte antigen (HLA)-restricted CTL clone (TAK-1) cells inhibited the formation of both CD34^+^CD59^+^ cells and CD34^+^CD59^-^ cell colonies. After co-culture with TAK-1 cells, the inhibition rate of CD34^+^CD59^-^ cell colony formation in 5 PNH patients was significantly lower than that of CD34^+^CD59^+^ cells, suggesting that WT1 peptide-specific and HLA-restricted cytotoxic T cells may play an important role in immune escape of PNH, which may be related to interferon-γ (43). These lines of evidence indicate that T lymphocytes may kill the GPI^+^ cells selectively, while GPI^-^ cells can escape from immunologic attack.

## The Possible Role of T Lymphocytes in the Pathogenesis of PNH

### T Lymphocytes May Play a Role Through GPI Molecule

**Figure 2:**
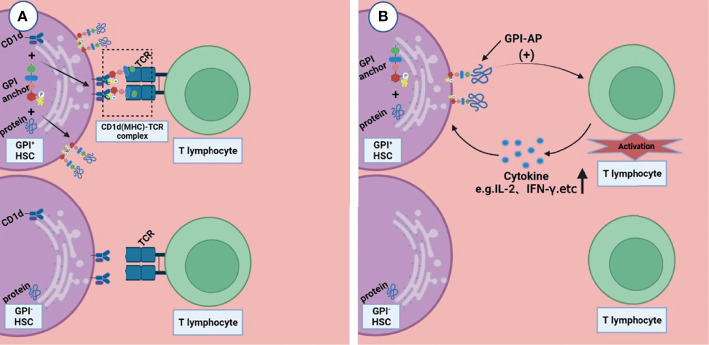
Two possible ways that T lymphocytes are involved in the pathogenesis of immune escape. **(A)** CD1d presents GPI molecule as antigen to T lymphocytes and then activates them, these T lymphocytes kill the GPI^+^HSC; however, the GPI^-^ HSC can escape from this attack. **(B)** GPI-AP mediates the activation of T lymphocytes and promotes the release of its cytokines (e.g., IL-2), then T lymphocytes kill HSCs expressing GPI-APs; however, HSCs do not express GPI-APs escape from this effect. Figures were created in BioRender.com.

Rotoli et al. proposed the hypothesis that in the pathogenesis of PNH, autoimmune T lymphocytes attack GPI^+^ HSC cells through GPI molecules, while GPI^-^ HSC cells can escape and survive (44). CD1d is a kind of MHC molecule that presents sugars/glycolipids that can induce T-cell immune responses; it is involved in the pathogenesis of a variety of diseases (45). Joyce et al. found that CD1d can present GPI anchor molecules (46). Thus, Karadimitris hypothesized that CD1d would present GPI molecule as antigen to T lymphocytes and then activate them. These T cells kill target cells that express GPI molecule; however, the PNH HSC, which lacks GPI molecules or has none at all, can escape from this attack (5). Gargiulo et al. analyzed the sequence of CDR-3 of TCR-β, and they found that identical or similar TCR-β chains were enriched in CD57^+^CD8^+^ T cells in PNH patients but not in normal controls, suggesting that auto-reactive T lymphocytes share a common target in most PNH patients (47). They also found that both exogenous and endogenous GPI molecules can activate CD1d restrictive T cells. This kind of T cells, known as the GPI specificity, CD1d-restrictive T cells, increased 10 times in PNH patients compared to normal control (48); this proof further supports the idea that T lymphocytes play the role of CTL by targeting GPI molecule by the CD1d(MHC)–TCR complex, and the GPI^-^ HSCs can escape from immune attack, although the specific mechanism is still unclear (**Figure 2A**).

### T Lymphocytes May Play a Role Through GPI-APs

Deckert et al. found that there are at least two pathways in the activation of T cells through CD59, resulting in the production of IL-2 (49); this suggests that GPI-AP as a co-stimulator mediates the cytotoxicity of T lymphocytes (**Figure 2B**). NT4.2 is a unique CD4^+^ T cell clone isolated by Nakao et al. from the bone marrow of AA patients, which strongly inhibited colony formation by HSPC (50). Takami et al. found that NT4.2 began to lyse LCL cells within 2 h and exhibited maximum cytotoxicity within 3 h. Anti-DR, CD2, CD3, CD58, and CD59 monoclonal antibodies (mAbs) were used to block the cytotoxic effect, and the results showed that all of the mAbs block the cytotoxic effect to the same degree, indicating that CD59 on the LCL surface, as a kind of GPI-APs, is required by T lymphocytes to produce a cytotoxic effect on target cells (51). CD4^+^ CTL produces cytotoxicity mainly *via* the Fas/FasL pathway (52). Ismall et al. studied 10 patients with PNH, and the expression of Fas in CD59^+^ cells were significantly higher than that in CD59^-^ cells in 3 patients (41). The Fas/FasL pathway is one of the apoptotic membrane receptor pathways (53). It is not difficult to speculate that T lymphocytes exerted cytotoxic effects through GPI-AP molecule *via* the Fas/FasL pathway, and CD59^+^ cells exhibited more apoptosis, while CD59^-^ cells could escape this cytotoxic effect.

## Perspectives

According to the results of the present studies, *PIG-A* gene mutation is widely considered as one of the pathogenesis of PNH, but there are at least 20 genes that are involved in GPI biosynthesis in addition to *PIG-A*, and additional genetic changes occurring in *PIG-A*-mutant HSCs could give these HSC clones a benign growth advantage (2). *PIG-A* gene mutation alone cannot explain the survival advantage of PNH clones, and it is not the only difference between the PNH clone and the non-PNH clone. Some researchers believe that it is the immunity of abnormal T lymphocytes that may be involved in the pathogenesis of PNH. The occurrence of PNH is due to the escape of GPI^-^ HSCs from the T cell-mediated autoimmune attack on GPI^+^ HSCs, which is known as immune escape hypothesis. IST therapy towards T lymphocyte is effective in some patients with PNH. However, PNH patients are rare and IST or other treatments have only been applied in very few cases, and the idea of immune escape still lacks evidence. The specific role that T lymphocytes play is still unclear, which requires further study until it can be discovered. Fully understanding the role of autoimmunity in that T lymphocytes are involved in the pathogenesis of PNH may provide more new and accurate strategies in treatment, which may slow down the attack from T lymphocytes on HSCs without *PIG-A* gene mutation and improve the quality of life of patients.

## Author Contributions

ZS and XD contributed to the conception and design of the study. CL wrote this manuscript. ZS, XD, and HW revised this manuscript. All authors contributed to manuscript revision, read, and approved the submitted version.

## Funding

This study is supported by the grant (32100656 to XD) from the National Natural Science Foundation of China.

## Conflict of Interest

The authors declare that the research was conducted in the absence of any commercial or financial relationships that could be construed as a potential conflict of interest.

## Publisher’s Note

All claims expressed in this article are solely those of the authors and do not necessarily represent those of their affiliated organizations, or those of the publisher, the editors and the reviewers. Any product that may be evaluated in this article, or claim that may be made by its manufacturer, is not guaranteed or endorsed by the publisher.
